# Anti-Caking Coatings for Improving the Useful Properties of Ammonium Nitrate Fertilizers with Composition Modeling Using Box–Behnken Design

**DOI:** 10.3390/ma14195761

**Published:** 2021-10-02

**Authors:** Aleksandra Tyc, Dominik Nieweś, Ewa Pankalla, Marta Huculak-Mączka, Krystyna Hoffmann, Józef Hoffmann

**Affiliations:** 1 Department of Engineering and Technology of Chemical Processes, Faculty of Chemistry, Wroclaw University of Science and Technology, Wybrzeże Wyspiańskiego 27, 50-370 Wrocław, Poland; dominik.niewes@pwr.edu.pl (D.N.); marta.huculak@pwr.edu.pl (M.H.-M.); 2 Department of Research and Innovations, Grupa Azoty Zakłady Azotowe Kędzierzyn, S.A., Mostowa 30A, 47-220 Kędzierzyn-Koźle, Poland; ewa.pankalla@grupaazoty.com; 3 Department of Micro, Nano and Bioprocess Engineering, Faculty of Chemistry, Wroclaw University of Science and Technology, Wybrzeże Wyspiańskiego 27, 50-370 Wrocław, Poland; krystyna.hoffmann@pwr.edu.pl

**Keywords:** fertilizers, ammonium nitrate fertilizers, anti-caking coatings, Box–Behnken design, response surface methodology

## Abstract

Granular fertilizers (especially those based on ammonium nitrate (AN)) tend to agglomerate during storage. The aims of this research were to develop effective anti-caking coatings for ammonium nitrate fertilizers while improving the quality of fertilizers and to optimize the composition of effective anti-caking coatings. The influence of the composition of the prepared organic coatings on the effectiveness of preventing the caking of fertilizers was studied by response surface methodology (RSM) using Box–Behnken design (BBD). Additionally, the effect of the developed anti-caking agents on the quality of fertilizers was determined by measuring the crushing strength of the granules. The prepared coatings included fatty amine, stearic acid, surfactant, and paraffin wax. Gas chromatography–mass spectrometry (GC–MS) was used to analyze these coatings. The morphology of the fertilizers were examined by scanning electron microscopy (SEM). Composition studies, based on statistical assessment, showed the coating components had a varying influence on preventing the caking of fertilizers after granulation and after 30 days of storage. The results demonstrated that increasing the content of fatty amines and reducing surfactant in the composition of coating had positive effects on caking prevention. In this study, more effective and economically viable anti-caking coatings were developed. In addition, the present work could serve as a basis to further improve anti-caking coatings.

## 1. Introduction

At present, one of the main requirements of the fertilizer industry is achieving the stabilization of the final product under storage and preventing changes in its form as a result of changing environmental conditions. The same demand is also faced by the chemical, pharmaceutical, and food industries [1]. The caking of various types of products occurs due to internal factors (e.g., chemical composition, hygroscopicity, water content, mechanical strength, and phase transformation) and external factors (e.g., moisture and air temperature, storage time, and pressure exerted during storage) [2,3,4,5,6,7]. Surface modification may reduce the impact of the adverse phenomena that the granules of nitrate fertilizers are subjected to [8,9,10].

The agglomeration of ammonium nitrate fertilizers (AN) are due to its physicochemical properties, which include high hygroscopicity, phase transformation, and explosive tendency (after simple treatment, improper storage, or use of additives) [11,12,13]. The main cause of ammonium nitrate fertilizers caking is connected with the phase change (IV→III) in ammonium nitrate occurring at the temperature of 32 °C [14]. This is when an increase in the volume of fertilizer granules occurs, and the consequence of this phenomenon is an increased pressure between the fertilizer granules that causes their deformation and an increase in the surface contact between them. Individual granules are connected to each other by “crystalline bridges” as water is involved in the process of phase change IV→III. Another reason for ammonium nitrate fertilizers caking is the presence of admixtures, hygroscopicity, and physical water content in the fertilizer. These factors are strongly interrelated. Some of the water in the granules forms hydrates, while some of the water that is free forms a thin layer of saturated solution on the surface of the fertilizer. In addition, under the action of surface tension forces, the ammonium nitrate solution present in the granule migrates towards the contact points between the granules with the formation of liquid bridges between them, which after evaporation become crystalline bridges [7,15]. Additionally, counteracting caking (depending on the composition of anti-caking agents) may protect nitrate fertilizers against large-scale uncontrolled explosions [16,17].

Initially, the method to prevent AN fertilizers from caking was to powder the finished product with inert dust (dolomite, diatomaceous earth, among others) and/or spray it with a surfactant and add inorganic salts or nucleating agents as stabilizers during fertilizer manufacturing. However, these methods were not very effective and additionally caused intense dusting [11,15,18]. Coating the surface of fertilizer granules with anti-caking coatings is a common practice today. The agents used aim to reduce or eliminate the influence of factors that cause fertilizer caking. These agents are usually characterized by the following effects: forming a hydrophobic barrier to the granule surface, inhibiting nucleation and crystallization, modifying crystallization, lowering surface tension, and reducing the strength of crystalline bridges [15]. Coatings intended to act as anti-caking agents and to improve the quality of the fertilizers have a low degree of coverage (about 0.1% by weight) [15,19]. This is significantly less compared to controlled-release or slow-release fertilizers, where fertilizer coating rates are approximately 3–20% by weight [20,21,22,23,24,25,26,27,28,29]. Commercial anti-caking agents used for fertilizers are based on petroleum feedstocks, such as slack wax and/or mineral oil, as the main bulk ingredient. The most important component, however, is a primary fatty amine and sometimes a secondary and tertiary fatty amine. The used amines can have different hydrocarbon chain lengths, but most often in the C_12_–C_18_ range [15]. Moreover, research on loose AN shows that the use of stearic acid and cetyl alcohol significantly reduces its hygroscopicity, which also reduces the caking of pure AN [30]. In addition, spray-dried particles consisting of AN, potassium nitrate, and a polymer (e.g., carboxymethylcellulose salt, polyvinyl alcohol, and styrene-butadiene latex) are effective in moisture proofing [31]. Thus, these substances can also prevent the agglomeration of nitrate fertilizer granules. Recent studies show that the hygroscopicity of nitrate fertilizers can be minimized by introducing additives such as silicic acid, calcium lignosulfonate, and sodium silicate into the fertilizer granules at the granulation stage [32,33]. 

The review indicates that further research is needed to develop new anti-caking agents, which will counteract the agglomeration of AN fertilizers and improve the physical parameters of the granules. There are no published studies showing the effect of individual ingredients on caking efficiency, optimization of anti-caking coating compositions, or the effect of coatings on fertilizer quality. The conducted research will allow us, in the following stages, to select new raw materials for coatings in order to make them more environmentally friendly. In particular, it will allow the replacement of paraffin and mineral oils, which form the basis of the currently used coatings, with other environmentally friendly raw materials.

After preliminary studies, it was found that combining a fatty amine with a fatty acid with a similar hydrocarbon chain length provides a good anti-caking effect. The proposed composition of anti-caking agents was enriched with surfactant. This allowed us to connect the positive effects of various components in one anti-caking composition. The increased effectiveness of such coatings is due to the presence of two different strong polar groups (an amine group and a carboxyl group) and a long hydrocarbon chain (the hydrophobic part). Amphiphilic compounds in anti-caking formulations, such as fatty amines and fatty acids, demonstrate the effect of lowering the interfacial energy of the crystal surface and solution, thus leading to the blocking of crystal nuclei. 

For determining the optimal composition of anti-caking agents, statistical methods can be used. The specific methodology is related to the number of independent variables and the expected results. The response surface methodology (RSM) allows establishing the dependencies between the independent variables, namely, process conditions or product composition, and the outcomes of the experiments. Recently, a modified model based on the Ginstling–Brounshtein model was developed to describe hydroxyapatite synthesis for different suspension concentrations [34]. In another study, ultrafast and green synthesis, by coprecipitation, of iron oxide-based magnetic nanoparticles was carried out using design of experiments (DOE) considering two main factors: the amplitude (energy) of the ultrasonic probe and the sonication time [35]. Depending on the complexity of the processes and the expected results, various experimental designs could be used. For more complex processes, where the results depend on many input variables, the Plackett–Burmann design (PBD) could be used. It is usually characterized by full saturation and enables determining significance levels. PBD is typically used to describe results that depend on many independent variables [36,37]. Another type of fractional plan can be used for a more accurate statistical evaluation. The most frequently used method is the Box–Behnken design (BBD), which allows us to consider not only the influence of the linear and square effects of the changes in the values of the independent variables, but also the influence of their linear interaction. With BBD, it is possible to generate three-dimensional plots that describe the influence of the values of the process parameters on the experimental results, as well as determine the effect estimates for the input experimental parameters and their interactions [38,39,40,41]. In a recent study, using the Box–Behnken design, the synthesis of silver nanoparticles from purple heart plant leaves extract [42], the extraction conditions of natural low-methoxyl pectin [43] and the HPLC separation of fluoroquinolones were optimized [44]. 

## 2. Materials and Methods

### 2.1. Materials

Uncoated AN fertilizers were obtained from their manufacturer (GA ZAK S.A., Kędzierzyn-Koźle, Poland). The tested AN fertilizer contained 32% nitrogen (N) including 16% nitric nitrogen and 16% ammoniacal nitrogen and consisted of a mixture of ammonium nitrate with calcium carbonate and magnesium carbonate (filler—dolomite). The fertilizer was produced by mechanical granulation method. At least 92% of the particles in the fertilizers were within the size range of 2–5 mm. Paraffin wax was purchased from Paraffin Wax (Międzyrzec Podlaski, Poland). Stearic acid (95%) was purchased from Merck (Darmstadt, Germany). Primary fatty amine (C_16_–C_18_) was procured from DHW Deutsche Hydrierwerke GmbH Rodleben (Dessau-Roßlau, Germany). The surfactant used contained mainly octanamide, and N,N-bis(2-hydroxyethyl) obtained from Brenntag (Essen, Germany). Chloroform with CZDA purity was acquired from Avantor Performance Materials Poland S.A. (Gliwice, Poland). 

### 2.2. Coating Process

A series of fertilizers with different coatings was prepared at the laboratory scale. The coating was prepared by mixing hot (65 ± 3 °C) paraffin wax with stearic acid, fatty amine, and surfactants in various proportions. The raw AN fertilizers were then sprayed with the prepared coatings in a rotary drum with inner vanes. The prepared coating (1.15 g) dissolved in 5 mL of chloroform was applied per kg of fertilizer. To spray the solution to cover the fertilizer granules, a sprinkler was used with a connected air supply hose to obtain a slight mist and direct the spray to the interior of the running drum with the fertilizer placed inside. The applied temperature (22 °C) and coverage were based on previous studies [45]. On an industrial scale, at the production facility, the anti-caking agent is sprayed at 70 °C directly onto the fertilizers (without the use of solvent). This eliminates the use of solvent to coat nitrate fertilizers with anti-caking coatings.

### 2.3. Analysis of Fertilizers

In this study, we focused on the effectiveness of organic coatings in preventing the caking of AN fertilizers. The effect of the composition of anti-caking agents on the caking prevention efficiency in granular AN fertilizers was estimated by statistical methods. The experimental points were defined using BBD for three input variables, where each had three levels denoted: –1, 0, and +1. The independent variables of the study and their values for the three levels are listed in Table 1. The tested anti-caking agents had four components, but only three independent component values. These values were defined as the content of the last component, paraffin wax, which served as a filler (to fill the coating mixtures up to 100% by weight). BBD analysis was performed using Statistica 13.3 software (Statsoft, Poland).

**Figure 1:**
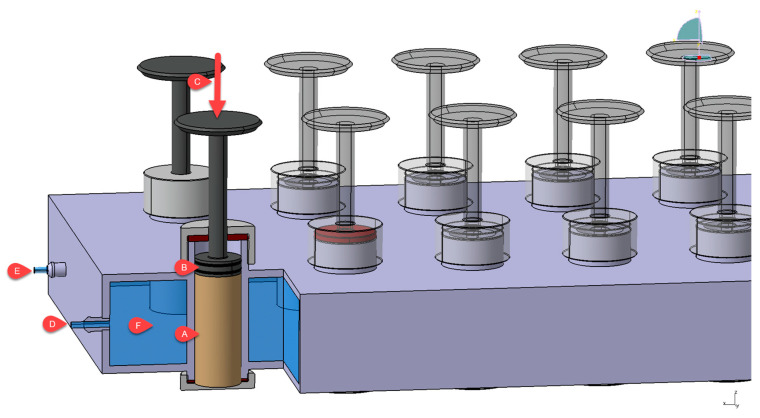
Scheme of the device used for delivering the temperature shocks under the load (A—sample chamber filled with fertilizer; B—piston; C—load; D—water inlet; E—water outlet; F—water circulation chamber).

The effectiveness in preventing caking (EPC) was examined by measuring the force needed to crush the clumped uncoated and coated fertilizers (using a vertical automatic crusher—IMADA), which were previously subjected to seven thermal cycles under a load (5 kg). One thermal cycle involved holding the sample for 1 h at 40 °C and then for 1 h at 20 °C. Figure 1 shows the device used for delivering the temperature shocks under the load. The EPC parameter was calculated based on the following formula [46]:

(1) $$\mathrm{EPC}\;\left(\%\right)=\frac{F_{o}-F}{F_{o}}\cdot 100\%,$$

where EPC is the effectiveness in preventing caking [%]; F_o_ is the force needed to crush the uncoated fertilizers [N]; and F is the force needed to crush the coated fertilizers [N]. 

EPC was determined after the granulation process (Y_1_) and after one month of storage (Y_2_). All samples were prepared and analyzed in triplicate. The results are presented as mean ± standard deviation. 

All fertilizer samples were stored under the same conditions (temperature 22 °C and humidity 45%) and the fertilizers were piled in the warehouse in the form of cones of the same size (piled 3 kg of fertilizer). A storage time of 30 days is generally sufficient to check the fertilizer’s tendency to cake after thermal cycles have been applied to the fertilizer. This has been determined experimentally and is subject to company standards in this area.

The mechanical properties of the prepared coated and uncoated fertilizers were determined by the measurements of crushing strength (CS) using Erweka GmbH. For each sample, 20 parallel measurements were performed (the diameters of the granules were 3.00–4.00 mm) and the mean value of the crushing strength was then calculated. 

The morphology of coated and uncoated AN fertilizers was examined using scanning electron microscopy (SEM; Carl Zeiss Microscopy EVO MA 15, Germany). Before the analysis, the granules were coated with a gold layer (in a vacuum atomizer).

### 2.4. Analysis of Coatings

Gas chromatography–mass spectrometry (GC–MS) analysis was performed to determine the most effective coating (or the most effective anti-caking agent). The analysis was carried out using a 7890 B GC system with a flame ionization detector (5977 MSD, Agilent Technologies) for simultaneous detection. Samples for analysis were dissolved in chloroform, and 1 µL of the samples was injected into a column (30 m × 0.25 mm × 0.25 µm; HP5 MS) with a 4:1 split (320 °C) at a constant flow rate (3 mL/min). The temperature program used was 120 °C (3 min), 10 °C/min–210 °C (6 min), and 10 °C/min–300 °C (23 min) [45].

## 3. Results and Discussion

### 3.1. Analysis of Fertilizers

Based on the BBD for three independent values, 13 compositions of anti-caking agents were tested. Fifteen experiments were performed according to the experimental matrix, and the last three experimental points were characterized by the central point of the Box–Behnken experimental space. Repeating the experiments for central points improved the resolution of the generated matrix. Due to the repetitions, the experimental error could be determined and the nonlinearity of the generated plan could be verified. Table 2 lists the points of the experimental matrix created according to BBD and the responses for these points. Independent variables were marked according to Table 1. The dependent variables for the experiments were the EPC results for fertilizer after granulation (Y_1_) and after 30 days of storage (Y_2_). The responses for each of the experimental points were defined three times for obtaining representative EPC values. The values of the final responses were expressed as mean ± standard deviation of the triplicate samples.

**Figure 2:**
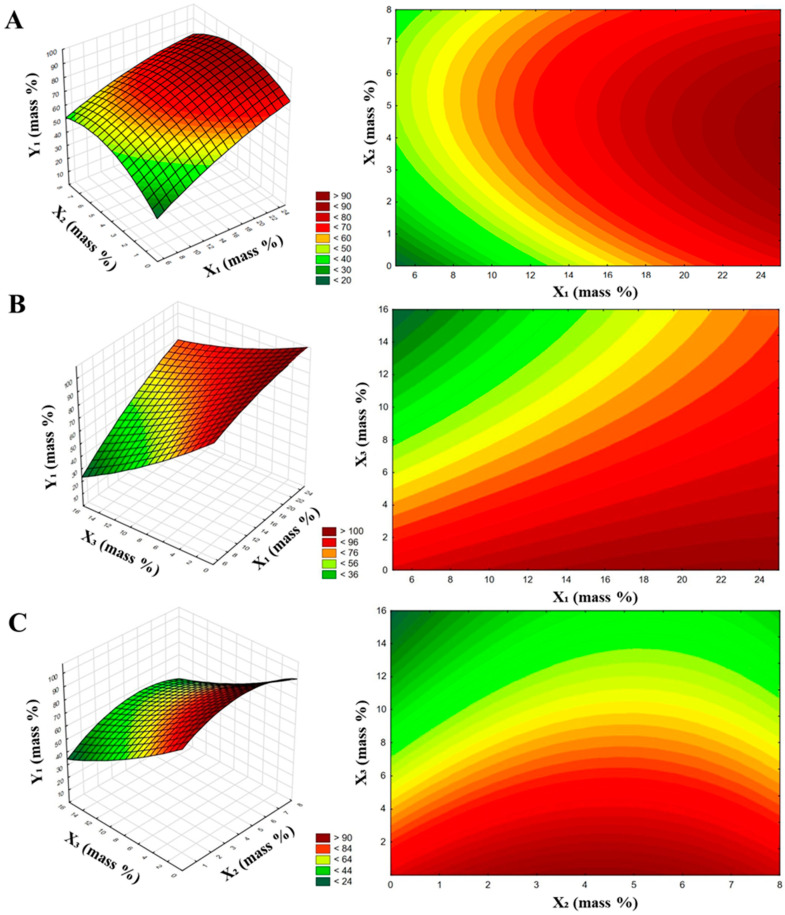
Response surface charts with contour charts representing the influence of the composition of coating agents on the effectiveness of caking prevention in fertilizers after granulation: (**A**) effect of fatty amine and stearic acid contents; (**B**) effect of fatty amine and surfactant contents; and (**C**) effect of stearic acid and surfactant contents.

**Figure 3:**
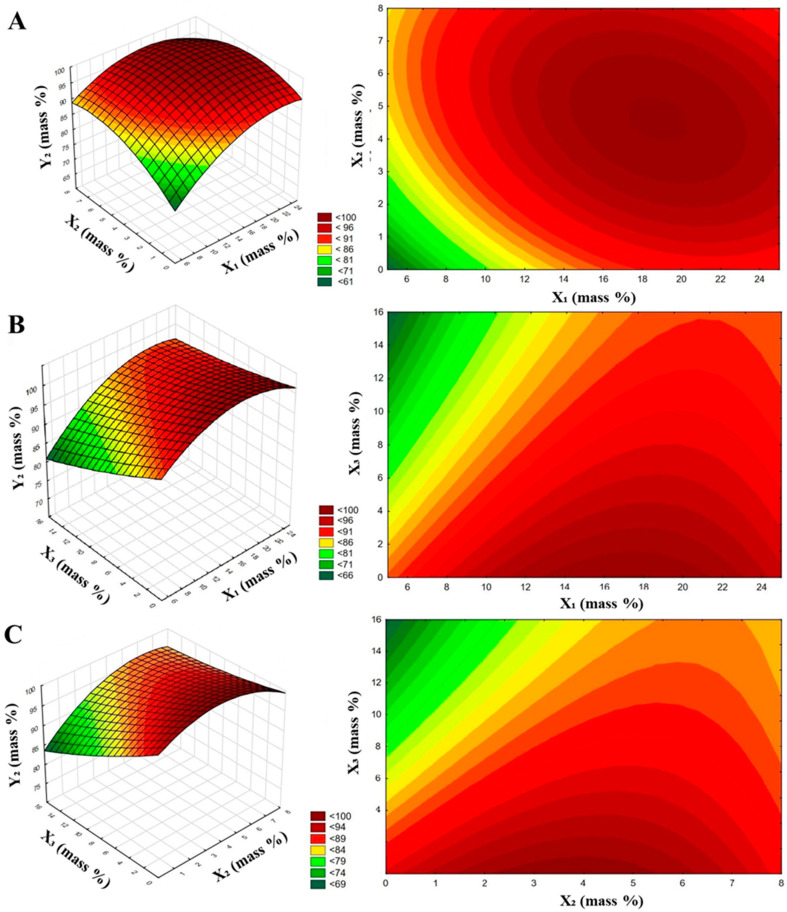
Response surface charts with contour plots representing the influence of the composition of coating agents on the effectiveness of caking prevention in fertilizers at 30 days of storage: (**A**) effect of fatty amine and stearic acid contents; (**B**) effect of fatty amine and surfactant contents; and (**C**) effect of stearic acid and surfactant contents.

Based on the results presented in Table 2, a statistical assessment was made for analyzing the effect of the composition of coating agents on the prevention of caking of granulated fertilizers. The interpretation consisted of two parts. The first determined the relationship between the composition of the coating agents and EPC parameter of the coated fertilizers. Figure 2 and Figure 3 show the response surface plots and contour charts. The three-dimensional plots describe responses as a function of two independent variables. For each of the figures, the third independent variable was constant and defined by a middle-level value, marked in Table 1 as 0.

On comparing the composition of anti-caking agents and its impact on the EPC parameter for fertilizers after granulation and 30 days of storage, it was observed that for freshly granulated AN fertilizers increasing the fatty amine content and decreasing the surfactant content improved the EPC. For the plots presented in Figure 2, we could not obtain the extremum for the values of the independent variables under BBD. A high density of wide-range contours, as seen in the plots in Figure 2A–C, implies a strong correlation between the content of fatty amine and surfactant and the EPC parameter.

From Figure 3, it can be observed that the EPC results for granules after 30 days of storage as a function of the composition of the coating agents were more complex than for the freshly granulated fertilizer. This is evidenced by the more curved contours of the plots; the fitted function for describing the dependence between the EPC parameter and the composition of the anti-caking agents reveals more than just the linear effects of the independent variables. 

In the second part of the statistical assessment, we analyzed the effect estimates of the factors on the responses. For this purpose, a quadratic model with linear interactions between independent variables was used. The effect estimates of caking prevention for freshly granulated fertilizers and granules after 30 days of storage are listed in Table 3 and Table 4. 

The results of effect estimates for fertilizer after granulation (Y_1_), presented in Table 3, showed the linear effect of fatty amine (X_1_) and surfactant (X_3_) contents on EPC values. The *p*-value was significant (*p* < 0.05) only for these two effects, thereby confirming the validity of the proposed model. The effect for X_1_ was greater than zero. The positive effect of fatty amine content in the composition of coatings means that an increase in amine concentration in the anti-caking agents has a positive effect on caking prevention in fertilizers after granulation. On the other hand, the effect of surfactant content (X_3_) on the experimental results was negative, which implies a negative effect of increasing the surfactant concentration on the anti-caking performance of fresh granules. The values of the statistical significance factor for other effects were more than 0.05. Due to their insignificance, those effects were ignored. In addition, the insignificant effects were characterized by high standard errors, which were probably caused by noise. 

The effect estimates of parameters for caking prevention in fertilizer after 30 days of storage showed the influence of linear and some quadratic factors on the EPC parameter. The values of statistically significant factors for the five effects listed in Table 4 were less than 0.05. Based on the effect values for the significant factors, it can be stated that increasing fatty amine and stearic acid contents in the prepared coatings has a positive effect on the results of the experiments. For the linear as well as quadratic independent variables, the effects were more than zero. The effect of surfactant content was less than zero, as with the effect of freshly granulated fertilizer. The effect estimates presented in Table 4 do not include the significance of the linear interaction between the independent parameters on the EPC results. 

Based on the effect estimates presented in Table 3 and Table 4, it can be concluded that fatty amine content and surfactant content in anti-caking agents are significant factors for improving the efficiency of caking prevention. For granules after 30 days of storage, the relationship between the composition of the coating and EPC results is more complex. This is due to water migration in fertilizers after granulation, which may be an important noise parameter in the assessment of the efficiency of anti-caking agents, especially in the case of freshly granulated fertilizers.

Statistical analysis allowed us to create polynomial equations that describe dependent variables (Y_1_ and Y_2_) as a function of the composition of anti-caking agents.

(2) $$Y_{1}=67.86+18.06\cdot X_{1}+2.21\cdot X_{1}^{2}+5.01\cdot X_{2}+6.51\cdot X_{2}^{2}-25.10\cdot X_{3}\;-2.01\cdot X_{3}^{2}-4.42\cdot X_{1}\cdot X_{2}+9.65\cdot X_{1}\cdot X_{3}+3.25\cdot X_{2}\cdot X_{3}$$

(3) $$Y_{2}=92.09+5.07\cdot X_{1}+3.01\cdot X_{1}^{2}+2.75\cdot X_{2}+2.51\cdot X_{2}^{2}-5.20\cdot X_{3}\;-0.36\cdot X_{3}^{2}-3.13\cdot X_{1}\cdot X_{2}+2.52\cdot X_{1}\cdot X_{3}+3.17\cdot X_{2}\cdot X_{3}$$

The correlation coefficient (R^2^) is 90.51% for model (2) and 95.61% for model (3). The *p*-value for Equations (2) and (3) is equal to 0.0064 and 0.0052, respectively. The high values for the R^2^ and p factors testify to a quite good fit of the created models to the experimental data and the significance of the proposed equations. 

On the basis of the presented models, optimal contents of components for anti–caking agents were predicted. Estimates show the maximum level of anti–caking prevention of optimized compositions for fertilizer after granulation and after 30 days of storage. The results of the optimalization are presented in Table 5.

**Figure 4:**
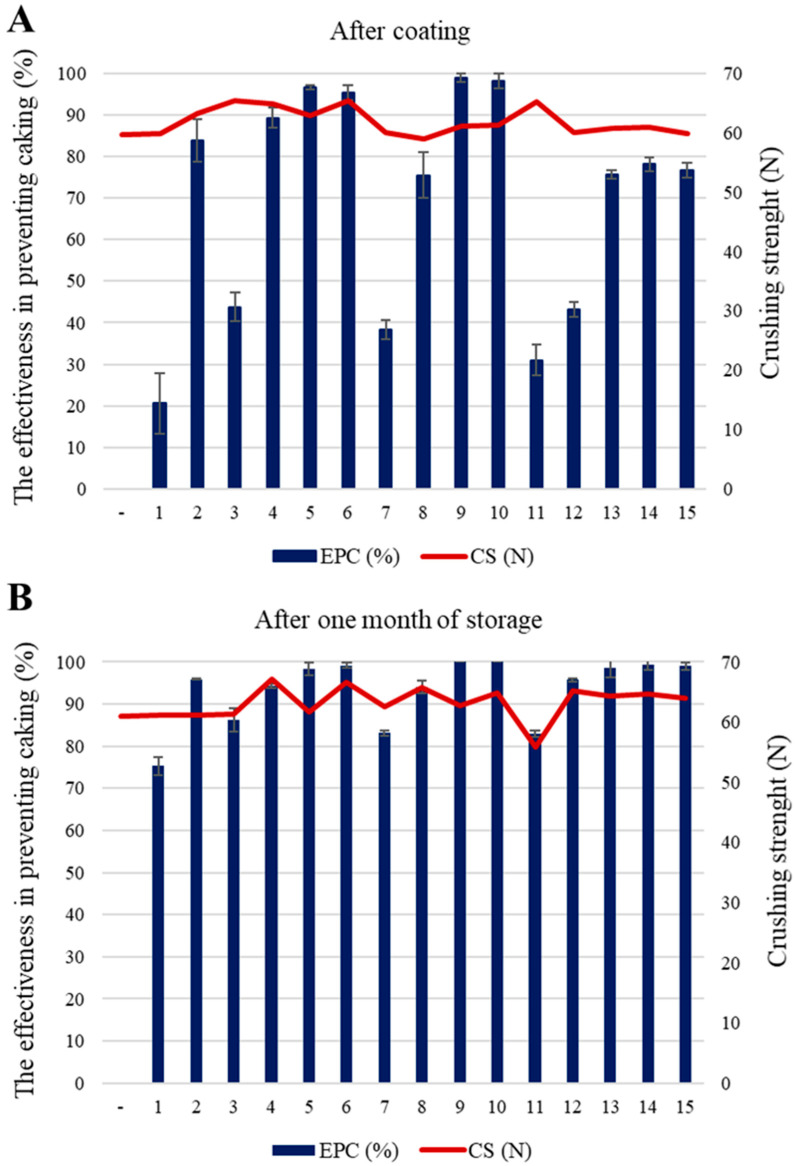
The effectiveness of anti-caking coatings and the crushing strength of granules: (**A**) after coating; and (**B**) after one month of storage.

Table 6 presents the results of the effectiveness of the developed anti-caking agents and the crushing strength of granules composed of ammonium nitrate fertilizers coated with the formulations and raw fertilizer (uncoated). Figure 4 shows graphically the dependence of the anti-caking effectiveness on the crushing strength of granules. The presented parameter was important due to the useful properties of AN fertilizer. It describes the resistance of granules to crushing during the storage and transport of the commercial product. The minimum crush strength of fertilizer granules is not standardized. However, the fertilizers used in this test have a crushing strength of granules (3–4 mm diameter) mostly above 50 N. There was no typical relationship between anti-caking performance and the crushing strength of the granules. Nevertheless, some formulations that showed very effective anti-caking properties were also found to increase the crushing strength of the granules compared to the raw fertilizer (formulations 5, 6, 9, and 10 were the best). Additionally, it can be seen that only one formulation deteriorated the crushing strength of the granules (formulation 8) immediately after coating, while this strength improved after one month of storage. Formulation 11 proved to be the least effective coating in terms of both effectiveness and granule crush strength. It is essential that, apart from being effective, the anti-caking agents do not deteriorate the quality of the fertilizers. In this respect, the most highly effective formulation turned out to be formulation 6, which protected the fertilizer from caking and ensured a very good quality of fertilizers. 

**Figure 5:**
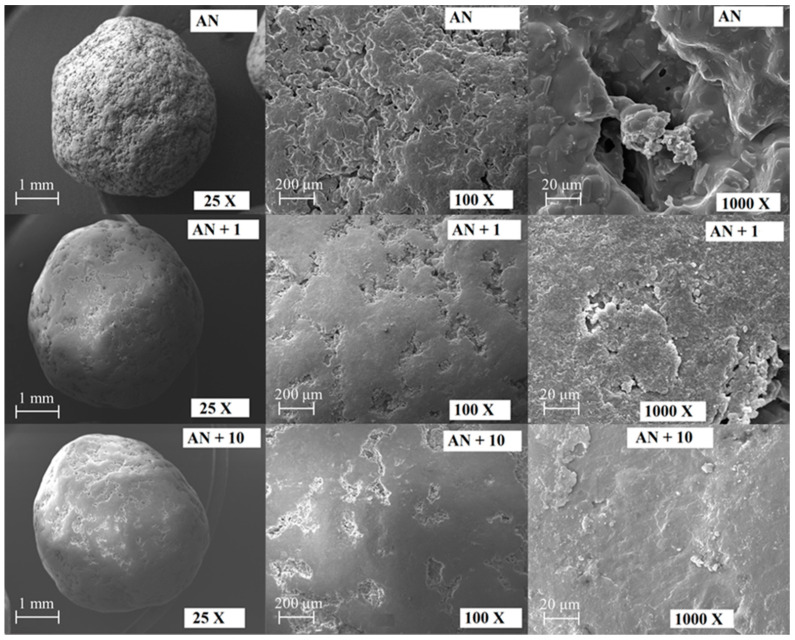
SEM images of uncoated AN fertilizer (AN) and AN fertilizers coated with 1 (AN + 1) and 10 (AN + 10) coatings. The image of each sample is shown at a magnification of ×25, ×100, and ×1000.

Based on the SEM images in Figure 5, the influence of the surface structure of AN fertilizer granules coated with coatings of different compositions on the prevention of caking was assessed. Anti-caking agents, marked in Table 2 as 1 and 10, were applied to AN granulated fertilizer in different compositions. Preparation 10 had more fatty amine and no surfactant. It showed one of the best EPC performances, while preparation 1 showed the worst EPC performance compared to all prepared organic coatings. The increase in fatty amine content and the elimination of the surfactant resulted in more effective coverage and reduced the water migration from the volume to the surface of the granules. This inhibited the formation of liquid bridges between the granules during storage. In addition, SEM analysis on the uncoated AN fertilizer showed that the coating setting affects the surface quality of the granule. The coating process improves the anti-caking properties of granules. It is particularly evident for the sample that was coated with composition number 10. SEM analysis showed a high degree of coverage for granules, which was found by comparison of roughness with uncoated granules. The positive effect of the coatings may be related to the creation of a barrier on the surface of granules. It prevents moisture absorption by hygroscopic AN. 

### 3.2. Analysis of Coatings

**Figure 6:**
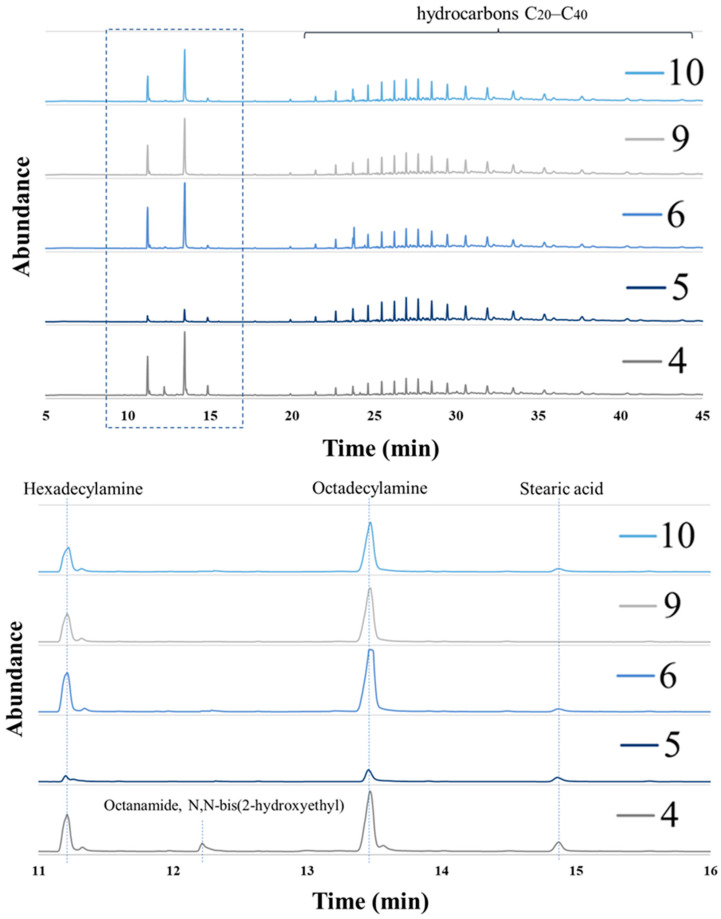
Chromatograms of the prepared coatings.

The purpose of GC–MS analysis was to determine the composition of anti-caking preparations and to check whether the compounds introduced into the developed preparations did not cause a chemical reaction when heated to 65 °C. The results of this analysis for the prepared coatings showing the best anti-caking efficiency are presented in Figure 6.

The obtained GC spectra showed that all of the five tested coatings (4, 5, 6, 9, 10) had in their composition only peaks originating from the ingredients introduced into the mixture. Coating 4 was the only one of the GC–MS-tested samples to have a surfactant in its composition, which consisted mainly of octanamide, N,N-bis(2-hydroxyethyl). It is only in this shell that the peak of this compound appeared in the chromatogram. The C16 amine and C18 amine peaks occurred in all coatings of varying intensity depending on the amount of a given compound incorporated into a particular formulation. Stearic acid was not included in formulation 9 as confirmed by the GC–MS analysis (no peak at this point). These compounds were identified both by comparison with the MS database and GC–MS spectra obtained for the individual raw materials used in the preparation of coatings. The peaks for the individual compounds are marked in the chromatograms (Figure 6). Those with a retention time of 20–40 min were derived from the slack wax used to develop the test preparations. These are hydrocarbon peaks with a hydrocarbon chain length of C20–C40. Only this area in the chromatogram is not identical for the intensity of the individual slack hydrocarbons, due to the heterogeneity of slack wax.

## 4. Conclusions

The aims of the present study were to develop effective anti-caking coatings for ammonium nitrate fertilizers while improving the quality of the fertilizers and to optimize the composition of effective anti-caking coatings. Statistical assessment of the relationship between the contents of the three components in anti-caking agents and the EPC value showed that increasing the fatty amine content and decreasing the surfactant content have a positive effect on the EPC parameter for fertilizers immediately after granulation and after 30 days of storage. Fatty amine turned out to be of key importance due to its amphiphilic structure, thanks to which it effectively reduces the interfacial energy of the crystal surface and the solution, thus leading to the blocking of crystal nuclei and reducing the tendency of fertilizers to cake. Additionally, the effect of anti-caking effectiveness on the crushing strength of the granules was studied. It showed that only one developed formulation slightly deteriorated the crushing strength of the granules after coating and after one month of storage. The remaining preparations improved the crushing strength of the granules while preventing caking. SEM images showed the association between the efficiency of coverage by anti-caking agents and their composition. The influence of the composition of the coatings on the EPC performance was found to be more complex for granulated fertilizers after 30 days of storage than for fertilizers after granulation, as it is associated with water migration from the volume to the surface of the granules just after granulation. In this study, several good anti-caking agents were developed and the most highly effective coating was formulation 6 (containing 25% by weight of amine and 4% by weight of stearic acid), which had high anti-caking efficiency and increased the crushing strength of the granules (both after coating and after one month of storage).

## Figures and Tables

**Table 1:** Levels and independent factors for the composition of anti-caking agents.

Levels	Independent Parameters		
	Fatty Amine Content (X_1_) (Mass %)	Stearic Acid Content (X_2_) (Mass %)	Surfactant Content (X_3_) (Mass %)
−1	5	0	0
0	15	4	8
1	25	8	16

**Table 2:** Results for the experimental points created according to the BBD. The last three points were marked as central for the experimental space.

Run	Independent Variables			Responses	
	X_1_	X_2_	X_3_	Y_1_	Y_2_
1	5	0	8	20.6 ± 7.3	75.3 ± 2.1
2	25	0	8	83.8 ± 5.0	95.9 ± 0.2
3	5	8	8	43.8 ± 3.4	86.2 ± 2.7
4	25	8	8	89.3 ± 2.5	94.3 ± 0.6
5	5	4	0	96.6 ± 0.5	98.3 ± 1.6
6	25	4	0	95.2 ± 1.8	99.2 ± 0.7
7	5	4	16	38.3 ± 2.4	83.1 ± 0.7
8	25	4	16	75.5 ± 5.4	94.1 ± 1.5
9	15	0	0	98.9 ± 1.1	100 ± 0
10	15	4	0	98.1 ± 1.7	100 ± 0
11	15	0	16	31.0 ± 3.7	83.0 ± 0.8
12	15	8	16	43.2 ± 1.8	95.7 ± 0.3
13	15	4	8	75.6 ± 1.1	98.6 ± 2.4
14	15	4	8	78.1 ± 1.6	99.3 ± 1.2
15	15	4	8	76.7 ± 1.8	99.0 ± 0.9

**Table 3:** Effect estimates for the effectiveness of caking prevention of fertilizers after granulation (Y_1_).

Parameter	Effect	Standard Error	*p*-Value	Remarks	Confidence Interval	
					−95%	+95%
**X_1_**	**36.12**	**9.78**	**0.0141**	**significant**	**10.99**	**61.26**
X_1_^2^	4.41	7.20	0.5665	not significant	−14.08	22.91
X_2_	10.02	9.78	0.3522	not significant	−15.11	35.16
X_2_^2^	13.01	7.20	0.1303	not significant	−5.48	31.51
**X_3_**	**−50.20**	**9.77**	**0.0037**	**significant**	**−75.33**	**−25.07**
X_3_^2^	−4.01	7.20	0.6011	not significant	−22.51	14.48
X_1_∙X_2_	−8.85	13.83	0.5503	not significant	−44.39	26.69
X_1_∙X_3_	19.30	13.83	0.2215	not significant	−16.24	54.84
X_2_∙X_3_	6.50	13.83	0.6580	not significant	−29.04	42.04

**Table 4:** Effect estimates for the effectiveness of caking prevention of fertilizers after 30 days of storage (Y_2_).

Parameter	Effect	Standard Error	*p*-Value	Remarks	Confidence Interval	
					−95%	+95%
**X_1_**	**10.15**	**1.93**	**0.0033**	**significant**	**2.60**	**7.55**
**X_1_^2^**	**6.02**	**1.42**	**0.0081**	**significant**	**1.19**	**4.83**
**X_2_**	**5.50**	**1.93**	**0.0356**	**significant**	**0.27**	**5.23**
**X_2_^2^**	**5.02**	**1.42**	**0.0165**	**significant**	**0.69**	**4.33**
**X_3_**	**−10.40**	**1.93**	**0.0029**	**significant**	**−7.68**	**−2.72**
X_3_^2^	−0.73	1.41	0.6289	not significant	−2.19	1.46
X_1_∙X_2_	−6.25	2.72	0.0702	not significant	−6.63	0.38
X_1_∙X_3_	5.05	2.72	0.1229	not significant	−0.98	6.03
X_2_∙X_3_	6.35	2.72	0.0671	not significant	−0.33	6.68

**Table 5:** Optimal compositions of anti-caking agents and predicted results of anti-caking prevention.

Type of Sample	Optimal Content of Components (Mass %)			Predicted Anti–Caking Prevention Efficiency (%)
	X_1_	X_2_	X_3_	
Fertilizer after granulation	18.87	4.43	0	100
Fertilizer after 30 days storage	22.08	4.41	0	100

**Table 6:** The effectiveness of anti-caking agents and the crushing strength of granules of AN fertilizers coated with the developed anti-caking coatings and of raw (uncoated) fertilizer.

Anti-Caking Coatings	Composition (X_1_:X_2_:X_3_) (Mass %)	After Coating		After One Month of Storage	
		EPC (%)	CS (N)	EPC (%)	CS (N)
-	-	-	59.7	-	61.0
1	5:0:8	20.6 ± 7.3	59.8	75.3 ± 2.1	61.2
2	25:0:8	83.8 ± 5.0	63.2	95.9 ± 0.2	61.2
3	5:8:8	43.8 ± 3.4	65.4	86.2 ± 2.7	61.4
4	25:8:8	89.3 ± 2.5	64.8	94.3 ± 0.6	67.1
5	5:4:0	96.6 ± 0.5	62.9	98.3 ± 1.6	61.6
6	25:4:0	95.2 ± 1.8	65.4	99.2 ± 0.7	66,7
7	5:4:16	38.3 ± 2.4	60.0	83.1 ± 0.7	62.5
8	25:4:16	75.5 ± 5.4	59.0	94.1 ± 1.5	65.7
9	15:0:0	98.9 ± 1.1	61.1	100 ± 0	62.7
10	15:4:0	98.1 ± 1.7	61.2	100 ± 0	64.8
11	15:0:16	31.0 ± 3.7	65.2	83.0 ± 0.8	55.8
12	15:8:16	43.2 ± 1.8	60.1	95.7 ± 0.3	65.2
13	15:4:8	75.6 ± 1.1	60.7	98.6 ± 2.4	64.3
14	15:4:8	78.1 ± 1.6	60.9	99.3 ± 1.2	64.7
15	15:4:8	76.7 ± 1.8	59.8	99.0 ± 0.9	64.0

## Data Availability

The data presented in this study are available on request from the corresponding author.
